# Comparing the Effects of Sitting Duration and Lateral Positioning on Hemodynamics and Neonatal Outcomes During Spinal Anaesthesia for Elective Caesarean Section

**DOI:** 10.7759/cureus.97895

**Published:** 2025-11-26

**Authors:** Manoj Kumar, Jay Brijesh Singh Yadav, Raghvendra Singh, Pramod Kumar Mishra, Rakesh Bahadur Singh

**Affiliations:** 1 Anaesthesiology, Uttar Pradesh University of Medical Sciences, Saifai, IND; 2 Anaesthesiology and Critical Care Medicine, Uttar Pradesh University of Medical Sciences, Saifai, IND

**Keywords:** cesarean section, hemodynamic stability, hyperbaric bupivacaine, patient positioning, spinal anaesthesia

## Abstract

Background: Spinal anaesthesia is widely used for caesarean sections. However, patient positioning during its administration can influence hemodynamic outcomes. This study compares the effects of different sitting durations and lateral positioning on hemodynamics and neonatal outcomes following spinal anaesthesia for elective caesarean section.

Methods: A total of 135 pregnant women scheduled for elective caesarean section under spinal anaesthesia were randomly divided into three groups of 45 each: Group S1 - Sitting for one minute, Group S2 - Sitting for two minutes, and Group L - Lateral position. Haemodynamic variables (heart rate, mean arterial pressure (MAP) and oxygen saturation (SpO_2_)), requirements for vasopressors, sensory and motor block levels, APGAR scores, patient satisfaction, and complications were recorded.

Results: After 10 minutes of spinal anaesthesia, MAP decreased significantly in Groups S1 and S2 compared to Group L (p < 0.05). Patients in Group L maintained better haemodynamic stability throughout the surgery. Sensory and motor block levels were comparable among all groups. The mean time to achieve the highest sensory block level was 4.20 ± 1.31 minutes in Group L, 6.73 ± 1.03 minutes in Group S1, and 6.82 ± 1.05 minutes in Group S2 (p < 0.05). Vasopressor requirement was significantly lower in Group L (6.43 ± 1.60 mg) compared to Groups S1 and S2 (14.25 ± 5.71 mg and 13.03 ± 5.57 mg, respectively) (p < 0.05). APGAR scores at one minute and five minutes were higher in Group L compared to Groups S1 and S2 (p < 0.05). Patient satisfaction scores were also significantly higher in Group L, with fewer complications compared to Groups S1 and S2.

Conclusion: The lateral position provided superior haemodynamic stability, higher patient satisfaction, and better neonatal outcomes compared to sitting positions during spinal anaesthesia for elective caesarean section.

## Introduction

Spinal anaesthesia is the most commonly employed technique for caesarean section due to its simplicity, rapid and reliable onset, and effectiveness in achieving adequate sensory and motor blockade [1,2]. Despite these advantages, it is frequently associated with significant maternal hypotension, which can adversely affect both maternal and fetal outcomes [3].

The sitting position is often preferred for administering spinal anaesthesia because it allows easier identification of anatomical landmarks and better patient cooperation. However, some studies suggest that it may be associated with a higher incidence of hypotension, likely due to the rapid cephalad spread of the local anaesthetic once the patient is positioned supine [4,5]. In contrast, the lateral position may allow a more gradual spread of the anaesthetic agent, potentially resulting in greater hemodynamic stability [6].

Various strategies have been introduced to minimise the hemodynamic fluctuations associated with spinal anaesthesia, and patient positioning during induction plays a crucial role in this regard. Both sitting and lateral positions are commonly used, each with distinct anatomical and physiological implications. Previous studies comparing these positions have reported differing results in terms of sensory block onset, ease of performance, and incidence of hypotension [7,8].

Recent research has also examined subtle variations within the sitting position itself. For instance, the duration for which a patient remains seated after intrathecal injection may influence anaesthetic spread and subsequent hemodynamic outcomes. However, limited data exist comparing specific durations, such as remaining seated for one minute versus two minutes before assuming the supine position.

Given this background, the present study aims to compare the effects of maintaining the sitting position for one minute, the sitting position for two minutes, and the lateral position on maternal hemodynamic parameters following spinal anaesthesia with hyperbaric bupivacaine in elective caesarean sections. Through this comparison, the study seeks to identify the optimal positioning strategy that minimises hypotension while maintaining adequate anaesthetic efficacy.

## Materials and methods

Aim

This study aims to evaluate and compare the effects of three different patient positions, sitting for one minute, sitting for two minutes, and the lateral position, on key hemodynamic variables and mephentermine requirements, following spinal anaesthesia with hyperbaric bupivacaine in patients undergoing caesarean sections.

Primary objective

The primary objective is to record and compare hemodynamic parameters, including mean arterial pressure (MAP), heart rate (HR), and oxygen saturation (SpO_2_), at various time intervals and positions.

Secondary objective

The secondary objectives were to assess the onset and peak levels of sensory and motor block, determine the total mephentermine requirement, evaluate neonatal APGAR scores, measure patient satisfaction scores, and document the incidence of complications and adverse effects.

Study design

A prospective, randomised controlled trial was conducted at Uttar Pradesh University of Medical Sciences (UPUMS), Saifai, India, from January 2020 to November 2021. The study was approved by the Institutional Ethical Committee, and written informed consent was obtained from all participants.

Sample size calculation

The sample size was calculated by using: $n = \frac{2 \times (Z_{1-\alpha/2} + Z_{1-\beta})^{2} \times \sigma^{2}}{d^{2}}$

Where power (1−β) = 80% → Z₁₋β = 0.84, confidence level = 95% → Z₁₋α/2 = 1.96, number of groups = 3. The calculated sample size was 45 patients per group (total = 135).

**Figure 1:**
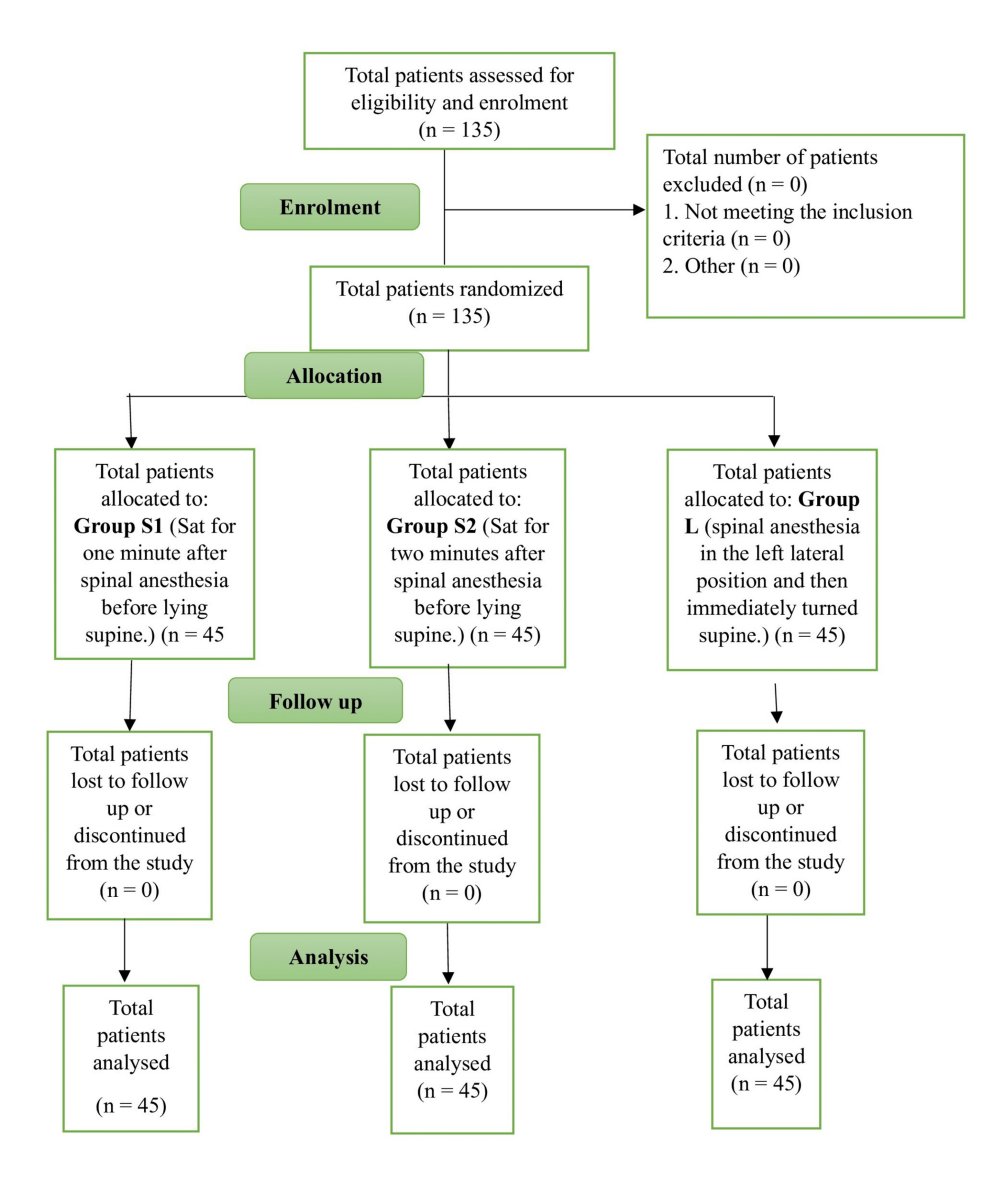
CONSORT flow diagram CONSORT: Consolidated Standards of Reporting Trials

A total of 135 pregnant women, aged 18-45 years and scheduled for elective caesarean sections, were enrolled and randomly allocated into three groups (n = 45 each) using sealed opaque envelopes. Group allocation was as follows (Figure 1):

Group S1 (Sitting Position for One Minute)

Patients remained seated for one minute after administration of the subarachnoid block before assuming the supine position.

Group S2 (Sitting Position for Two Minutes)

Patients remained seated for two minutes after administration of the subarachnoid block before assuming the supine position.

Group L (Lateral Position)

Patients received the subarachnoid block in the left lateral position and were immediately turned to the supine position afterwards.

Inclusion criteria

Inclusion criteria included pregnant females (gestational age 37-42 weeks) scheduled for elective caesarean section, aged 18-45 years, with American Society of Anesthesiologists (ASA) physical status II, and carrying a singleton pregnancy.

Exclusion criteria

Exclusion criteria included refusal to participate; known hypersensitivity to local anaesthetics; bleeding disorders; failed subarachnoid block requiring conversion to general anaesthesia; systemic disorders such as preeclampsia, chronic hypertension, diabetes mellitus, chronic obstructive pulmonary disease, cerebrovascular disease, cardiovascular disease, or psychiatric illness; pre-existing neurological deficits; severe hypovolemia; and any contraindications to neuraxial anaesthesia.

Anaesthetic technique

All participants received a standardised dose of 0.5% hyperbaric bupivacaine (2.5 mL) administered at the L3-L4 intervertebral space using a 26G Quincke’s spinal needle. Following drug administration, patients were positioned according to their allocated groups and given a left lateral tilt to prevent aortocaval compression.

Data collection

Hemodynamic Parameters

Systolic blood pressure (SBP), diastolic blood pressure, MAP, HR, and SpO_2_ were recorded at baseline (before anaesthesia), every minute for the first five minutes after anaesthesia, and subsequently every five minutes up to 30 minutes, followed by sensory block assessment.

Sensory block was evaluated bilaterally using the pinprick method, beginning immediately after positioning and repeated every minute until a T4 sensory level was achieved, at which point surgery commenced; sensory block was graded as 0 = normal sensation, 1 = loss of pinprick sensation (analgesia), and 2 = loss of touch sensation (anaesthesia). Motor block was assessed using the Modified Bromage Scale (MBS), where 0 = no motor block, 1 = inability to raise the extended leg but able to move the knees and feet, 2 = inability to raise the leg and move the knee but able to move the feet, and 3 = complete motor block.

Management of Hypotension

Hypotension was defined as a decrease in MAP >30% from baseline or SBP <90 mmHg. It was treated with intravenous mephentermine 6 mg bolus, repeated as necessary to maintain SBP >90 mmHg.

APGAR Score

The APGAR score was assessed at one and five minutes after birth to evaluate neonatal well-being based on five criteria: Appearance, Pulse, Grimace, Activity, and Respiration. Each category was scored from 0 to 2, with a total possible score of 0-10 (Table 1).

**Table 1:** APGAR score

Score	0 point	1 point	2 points
Appearance (Skin color)	Cyanotic/pale Over body	Peripheral cyanosis only	Pink
Pulse (Heart rate)	0	<100	100-140
Grimace (reflex irritability)	No response to stimuli	Grimace or weak cry when stimulated	Cry with stimulated
Activity (tone)	Floppy	Some flexion	Well flexed and resisting extension
Respiration	Apneic	Slow, irregular breathing	Strong cry

Patient satisfaction was assessed using a 7-point Likert verbal rating scale immediately after surgery and again at 24 hours, where 1 = extremely dissatisfied, 2 = dissatisfied, 3 = somewhat dissatisfied, 4 = undecided, 5 = somewhat satisfied, 6 = satisfied, and 7 = extremely satisfied.

Adverse Effects

Any adverse effects, such as nausea, vomiting, vertigo, bradycardia, or discomfort, were recorded and managed appropriately.

Statistical analysis

All data were analysed using IBM SPSS Statistics for Windows, Version 22 (Released 2013; IBM Corp., Armonk, New York, United States). Continuous variables were analysed using analysis of variance (ANOVA), while Chi-square tests were used for categorical variables. A p-value <0.05 was considered statistically significant.

## Results

The baseline characteristics of the participants in the three groups were comparable, with no statistically significant differences in age, body mass index (BMI), or baseline hemodynamic variables (p > 0.05) (Table 2).

**Table 2:** Demographic characteristics of the groups * One-way analysis of variance (ANOVA)

Characteristics	Group L	Group S1	Group S2	P-value
Age (years)	26.29±3.61	26.69±3.10	26.42±3.11	0.841*
Duration of surgery (minutes)	76.16±3.86	76.02±4.20	76.89±4.10	0.552*

Mean arterial pressure (MAP)

Group L maintained a more stable MAP compared to Groups S1 and S2, with fewer instances of hypotension. During the second minute, third minute, fifth minute, and 10th minute, fall in MAP was statistically significant when Group L was compared with Group S1 and Group S2 (p < 0.001). However, there was no significant difference observed between S1 and S2 at all time intervals except at the third minute (Table 3).

**Table 3:** Comparison of mean arterial pressure (MAP) at various time points among the groups ^#^ One-way analysis of variance (ANOVA); ^##^ Post-hoc Tukey test; * Statistically significant

Time interval (minutes)	Group L	Group S1	Group S2	P-value^#^	Group L vs. Group S1^##^	Group L vs. Group S2^##^	Group S1 vs. Group S2^##^
	Mean±SD	Mean±SD	Mean±SD				
Baseline	93.94±4.163	92.36±6.927	94.57±8.236	0.271	0.498	0.896	0.26
1	77.2 ±7.099	74.67±4.487	74.63±6.396	0.075	0.122	0.116	1
2*	81.3±7.288	60.59±4.747	61.16±3.916	<0.001*	<0.001*	<0.001*	0.875
3*	84.7±7.578	61.6 ±5.287	64.62±4.457	<0.001*	<0.001*	<0.001*	0.046
5*	87.27±5.992	75.28±6.231	73.91±4.105	<0.001*	<0.001*	<0.001*	0.465
10*	86.6±5.495	81.81±5.761	79.32±10.948	<0.001*	0.012*	<0.001*	0.289
15	84.24±5.474	82.4±5.37	81.51±8.917	0.156	0.409	0.141	0.806
20	87.74±4.891	86.11±6.49	86.43±9.996	0.549	0.556	0.682	0.978
25	88.22±5.483	87.39±5.521	86.16±9.568	0.387	0.844	0.358	0.692
30	88.11±5.784	88.24±5.663	87.05±8.098	0.648	0.995	0.729	0.673
40	87.15±6.626	88.9±5.4	86.51±8.159	0.233	0.446	0.899	0.226
50	88.01±5.242	88.83±5	86.99±6.613	0.308	0.774	0.669	0.276
60	88.53±4.477	88.89±4.92	87.11±6.951	0.279	0.947	0.451	0.285
70	88.51±4.904	89.9±5.14	86.33±8.289	0.029	0.553	0.232	0.022
80	78.32±19.144	80.94±20.056	87.47±4.955	0.436	0.859	0.403	0.627

During comparison among the groups, the mean HR was found to be less variable and statistically significant in group L versus Group S2 at the third minute (p = 0.002). During intergroup comparison, the mean values were not statistically significant among the groups (Table 4).

**Table 4:** Comparison of mean heart rate (beats/min) at various time points among the groups ^#^ One-way analysis of variance (ANOVA); ^##^ Post-hoc Tukey test; * Statistically significant

Time interval (minutes)	Group L	Group S1	Group S2	P-value^#^	Group L vs. Group S1^##^	Group L vs. Group S2^##^	Group S1 vs. Group S2^##^
	Mean ± SD	Mean ± SD	Mean ± SD				
Baseline	99.02±8.37	95.4±12.26	96.93±11.64	0.289	0.259	0.635	0.783
1	94.71±6.91	93.8±15.4	94.22±9.11	0.927	0.920	0.976	0.982
2*	91.56±5.63	95.36±12.5	94.2±11.39	0.203	0.190	0.444	0.855
3*	89.2 ±6.00	95.09±11.17	96.22±10.38	0.001*	0.010	0.002*	0.837
5*	87.51±6.15	92.80±9.95	93.00±9.95	0.005*	0.015	0.011	0.994
10*	87.96±6.27	85.67±9.79	91.8 ±9.22	0.016*	0.030	0.036	0.997
15	86.69±5.47	87.78±9.78	88.33±12.58	0.718	0.856	0.703	0.960
20	88.13±6.03	91.2 ±9.84	97.22±11.63	0.294	0.277	0.548	0.876
25	88.27±6.16	88.91±9.79	89.13±9.90	0.889	0.936	0.887	0.992
30	88.56±6.85	85.91±9.8	88.69±8.89	0.229	0.314	0.997	0.279
40	89.22±5.59	87.98±8.43	90.96±7.87	0.163	0.705	0.509	0.140
50	87.71±4.88	86.76±8.7	90.31±7.99	0.064	0.812	0.220	0.061
60	86.82±5.56	85.78±8.16	88.71±7.29	0.142	0.764	0.418	0.125
70	88.86±4.49	85.66±8.36	87.24±8.11	0.117	0.096	0.548	0.567
80	86.64±5.1	84.17±8.88	88.33±5.13	0.329	0.593	0.869	0.405

Oxygen saturation (SpO_2_)

SpO_2_ levels remained comparable among all the groups (p > 0.05).

Both peak sensory and motor levels were achieved earlier in Group L, followed by S1 and S2 later. The mean difference was statistically significant among the groups. During intergroup comparison between Group L versus S1 and S2, the mean difference was statistically significant (p < 0.001). However, the mean difference was comparable between groups S1 and S2 (p > 0.05) (Table 5).

**Table 5:** Comparison of time to achieve peak sensory level and peak motor block ^#^ One-way analysis of variance (ANOVA); ^##^ Post-hoc Tukey test; * Statistically significant

Parameters	Group L	Group S1	Group S2	P-value^#^	Group L vs. Group S1^##^	Group L vs. Group S2^##^	Group S1 vs. Group S2^##^
	Mean± SD	Mean± SD	Mean± SD				
Peak sensory level achieved (T4 level in minutes)	4.20±1.31	6.73±1.03	6.82±1.05	<0.001*	<0.001*	<0.001*	0.927
Motor block achieved (Modified Bromage Scale in minutes)	6.44±2.16	8.47±1.88	8.89±2.41	<0.001*	<0.001*	<0.001*	0.502

The total requirement of mephentermine was lower in Group L. There was a statistically significant difference in mephentermine requirement during comparison between Group L versus Group S1 (p < 0.001) and Group L versus Group S2 (p = 0.001) (Table 6).

**Table 6:** Comparison of mephentermine required among groups ^#^ One-way analysis of variance (ANOVA); ^##^ Post-hoc Tukey test; * Statistically significant

Parameter	Group L	Group S1	Group S2	P-value^#^	Group L vs. Group S1^##^	Group L vs. Group S2^##^	Group S1 vs. Group S2^##^
	Mean±SD	Mean±SD	Mean±SD				
Mephentermine required (in mg)	6.43±1.60	14.25±5.71	13.03±5.57	<0.001*	<0.001*	<0.001*	0.607

APGAR score in Group L was better in comparison to Groups S1 and S2. There was a significant difference in APGAR score among the groups both at one minute and five minutes, i.e., Group L versus Group S1 (p < 0.001) and Group L versus Group S2 (p = 0.001) (Table 7).

**Table 7:** Comparison of mean APGAR score between the groups ^#^ One-way analysis of variance (ANOVA); ^##^ Post-hoc Tukey test; * Statistically significant

APGAR score	Group L	Group S1	Group S2	P-value^#^	Group L vs. Group S1^##^	Group L vs. Group S2^##^	Group S1 vs. Group S2^##^
	Mean±SD	Mean±SD	Mean±SD				
1 minute	7.16±1.11	5.04±1.02	5.49±0.94	<0.001*	<0.001*	<0.001*	0.104
5 minutes	9.22±0.850	7.71±1.12	7.93±0.94	<0.001*	<0.001*	<0.001*	0.528

Patient satisfaction score was better in Group L, and the mean difference was significant among the groups (p < 0.05). During intergroup comparison, the mean difference in satisfaction scores was significant between Group L and Groups S1, S2. However, the comparable values were reported between Groups S1 and S2 (p = 0.189) (Table 8).

**Table 8:** Comparison of mean patient satisfaction score between the groups ^#^ One-way analysis of variance (ANOVA); ^##^ Post-hoc Tukey test; * Statistically significant

Parameter	Group L	Group S1	Group S2	P-value^#^	Group L vs. Group S1^##^	Group L vs. Group S2^##^	Group S1 vs. Group S2^##^
	Mean± SD	Mean± SD	Mean± SD				
Patient Satisfaction Score	5.62 ± 0.58	4.36 ± 0.68	4.09 ± 0.87	<0.001*	<0.001*	<0.001*	0.189

Complications such as nausea (N), vomiting (V), bradycardia (B), and shivering (S) among the groups were also compared and observed statistically as not significant among the groups. During intergroup comparison, Group L versus S1, Group L versus S2, and Group S1 versus S2, the mean values were observed to be statistically not significant (Table 9).

**Table 9:** Comparison of complications between the groups A Chi-square test was used; * Statistically significant.

Complications	Group L	Group S1	Group S2	P-value	Group L vs. Group S1	Group L vs. Group S2	Group S1 vs. Group S2
	N (%)	N (%)	N (%)				
Nausea and Vomiting (NV)	4 (8.9%)	8 (17.8%)	3 (6.7%)	0.207	0.353	1.000	0.197
Bradycardia	0 (0%)	3 (6.7%)	2 (4.4%)	0.234	0.242	0.494	1.000
Shivering	0 (0%)	3 (6.7%)	2 (4.4%)	0.234	0.242	0.494	1.000
Overall	4 (8.9%)	14 (31.1%)	7 (15.6%)	0.021*	0.016*	0.522	0.081

## Discussion

Spinal anaesthesia is widely used for caesarean delivery because of its rapid onset, dense neural blockade, and minimal risk of local anaesthetic toxicity. However, hypotension remains a common adverse effect in obstetric patients undergoing neuraxial anaesthesia. The haemodynamic changes following spinal anaesthesia occur abruptly, predisposing mother and foetus to complications associated with hypotension. In the present study, demographic characteristics were comparable among all groups (p > 0.05).

Sensory and motor blockade

The mean time to attain the peak sensory level (T4) was significantly shorter in Group L (4.20 ± 1.31 minutes) compared with Group S1 (6.73 ± 1.03 minutes) and Group S2 (6.82 ± 1.05 minutes) (p < 0.001). Similarly, the time to achieve complete motor block (MBS 3) was significantly lower in Group L (6.44 ± 2.16 minutes) compared with Group S1 (8.47 ± 1.88 minutes) and Group S2 (8.98 ± 2.41 minutes) (p < 0.001). Thus, both sensory and motor block onset occurred fastest in the lateral position, followed by Group S1, and slowest in Group S2.

These findings align with those of Achary et al. [6], who reported delayed sensory onset in the sitting position due to gravitational settling of hyperbaric bupivacaine, limiting cephalad spread. Kharge et al. [7] also observed higher sensory levels in the lateral position. Similarly, studies by Laithangbam et al. [4] and Shahzad and Afshan [8] demonstrated a quicker onset of motor block in the lateral position, although mean differences were statistically not significant. Prakash et al. [5] and Inglis et al. [9] also supported these results, reporting faster onset of sensory block in the lateral position without significant differences in maximum block height or motor block intensity.

Hemodynamic parameters

During monitoring, a greater increase in HR was observed in Groups S1 and S2 compared with Group L (p < 0.05), with comparable mean values between Groups S1 and S2. This suggests superior hemodynamic stability in the lateral position.

The greater hemodynamic fluctuations in the sitting position may be attributed to vasovagal responses, gravity-dependent venous pooling, reduced cardiac output, and orthostatic hypotension. Obasuyi et al. [10] reported a lower incidence of hypotension in the lateral group (34%) compared with the sitting group (56%). Similar findings were reported by Agrawal and Rawlani [11] and Ali et al. [12], who observed a higher rate of hypotension in the sitting position. Although Kharge et al. [7] suggested that preloading could mitigate haemodynamic variations, the lateral position remained more comfortable for patients.

APGAR score

APGAR scores differed significantly between groups (p < 0.001), with Group L exhibiting higher scores than Groups S1 and S2. Although Coppejans et al. [13] found similar APGAR scores across different positions, they noted higher umbilical artery pH in the sitting group, suggesting that neonatal outcomes may vary depending on the parameters assessed.

Mephentermine requirement

The mean mephentermine requirement was significantly lower in Group L (6.43 ± 1.60 mg) compared with Group S1 (14.25 ± 5.71 mg) and Group S2 (13.03 ± 5.57 mg) (p = 0.001). These results are consistent with the findings of Russell [14] and Chestnut et al. [15], who reported greater vasoconstrictor use in the sitting position owing to prolonged and more pronounced hypotension.

Patient satisfaction

Patient satisfaction was highest in Group L (5.62 ± 0.58), followed by Group S1 (4.36 ± 0.68) and Group S2 (4.09 ± 0.87), with statistically significant differences among groups (p < 0.001). This indicates greater comfort in the lateral position. Manouchehrian et al. [16], Shahzad and Afshan [8], and Kharge et al. [7] similarly reported that although spinal anaesthesia may be technically easier to perform in the sitting position, patients perceived the lateral position to be more comfortable.

Complications

Nausea and vomiting occurred most frequently in Group S1 (17.8%), followed by Group S2 (8.9%) and Group L (6.7%). Bradycardia requiring intervention was also more common in the sitting groups. Jackson et al. [17] and Yun et al. [18] attributed these complications to vasovagal reflexes and increased parasympathetic activity in the sitting position. Shahzad and Afshan [8] similarly reported a higher incidence of bradycardia in the sitting position, although the difference was not statistically significant.

Limitations

A key limitation of this study was the difficulty in blinding participants to their assigned positions. However, bias was minimised by blinding both the data collector and a second anaesthesiologist responsible for assessing participants’ clinical data. Maternal BMI was not recorded, which may have influenced haemodynamic responses, given the higher risk of hypotension in individuals with elevated BMI. Additionally, the study did not evaluate the incidence of post-dural puncture headache (PDPH) or bloody spinal taps, which are generally more common in the sitting position.

## Conclusions

The findings of this study demonstrate that the lateral position provides superior hemodynamic stability compared with both sitting positions for one minute and two minutes, thereby reducing the incidence of maternal hypotension and decreasing the requirement for vasopressor support. Furthermore, the onset of peak sensory and motor blockade was significantly faster in the lateral position. Patients in the lateral position group required less mephentermine and had higher neonatal APGAR scores, indicating more favourable maternal and neonatal outcomes.

Overall, these results suggest that performing spinal anaesthesia in the lateral position offers notable advantages in terms of cardiovascular stability and rapid onset of block. Although the sitting position remains useful - particularly when anatomical landmarks can be easily identified or when precise control of block height is required, such as in emergency or technically challenging situations - it is not always necessary in routine practice. The lateral position may be the preferred choice when minimising hemodynamic fluctuations is a primary concern.
